# Abnormal response to chronic social defeat stress and fear extinction in a mouse model of *Lynx*2-based cholinergic dysregulation

**DOI:** 10.3389/fnins.2025.1466166

**Published:** 2025-04-01

**Authors:** Kristin R. Anderson, Peter J. Rogu, Talulla B. Palumbo, Julie M. Miwa

**Affiliations:** Department of Biological and Chemical Sciences, Bethlehem, PA, United States

**Keywords:** nAChR, Lynx2, LYPD1, social interaction, anxiety behavior, nicotinic acetylcholine receptors, CSDS, defeat stress

## Abstract

Nicotinic receptor signaling is influential in modulating appropriate responses to salient stimuli within a complex environment. The cholinergic neurotransmitter system drives attention to salient stimuli such as stressors, and aids in orchestrating the proper neural and behavioral responses. Dysregulation of this system, however, has been implicated in altered anxiety regulation and mood disorders. Among the multiple layers of regulation are protein modulators such as Lynx2/Lypd1, which provides negative nicotinic acetylcholine receptor regulation within anxiety-related circuits, such as the amygdala and medial prefrontal cortex, among other brain regions. Mice null for Lynx2/Lypd1 (Lynx2 KO) show elevated basal anxiety-like behavior in tests such as elevated plus maze, light-dark box and social interaction assays. Here, we queried how a line predisposed to basal anxiety-like behavior would respond to specific stressors, using validated models of experiential-based affective disorders such as fear extinction, acute and chronic social defeat stress assays. We discovered that Lynx2 KO mice demonstrate an inability to extinguish learned fear during fear extinction tests even during milder stress conditions. In social defeat studies, contrary to our predictions, the Lynx2 KO mice switched from a socially avoidant phenotype (which could be considered susceptible) before defeat to a social approach/resilient phenotype after defeat. Consistent with reports of the inverse relationship between resilience and BDNF levels, we observed reduced BDNF levels in the VTA of Lynx2 KO mice. Furthermore, we provide evidence for the functional role of α7 nicotinic receptor subtypes by phenotypic rescue of fear extinction and social defeat phenotypes by MLA antagonism of α7 nicotinic acetylcholine receptors, or by crossing with α7 nicotinic acetylcholine receptor null mutant mice. A stable physical interaction between LYNX2 and α7 nAChRs was observed by co-immunoprecipitation of complexes from mouse amygdalae extracts. Together, these data indicate that responses to specific stressors can become aberrant when baseline genetic factors predispose animals to anxiety dysregulation. These studies underscore the critical nature of well-regulated nicotinic receptor function in the adaptive response to environmental stressors.

## Introduction

The stress response is an adaptive suite of physiological and psychological changes that helps an individual to galvanize appropriate responses to threatening situations (Robinson et al., 2019). If not regulated properly, however, individuals can develop persistent, pervasive, and generalized anxiety or fear in the form of an affective disorder that is detrimental to quality of life (Grogans et al., 2023) and which can lead to maladaptive damage in the long-term and comorbidities such as depression (Maron and Nutt, 2017; Schneiderman et al., 2005). The capacity to moderate one’s response to stressors—to balance adaptive protection in the short term and maladaptive changes in the long term—is critical as disorders involving anxiety and fear are among the most prevalent mental disorders, affecting 33.7% of US adults at some point in their lives (McEwen, 2017; Stein et al., 2017; Grogans et al., 2023; Kessler et al., 2012). Despite the high prevalence of affective disorders, current treatments do not meet the full need, highlighting the utility of further understanding the complex underpinnings of anxiety to inform the development of effective treatments.

The cholinergic system is an important modulatory neurotransmitter system involved in a number of adaptive behaviors. Nicotinic acetylcholine receptors (nAChRs) of the cholinergic system have been shown to regulate activity in anxiety, fear, and depressive-related circuits and related behaviors in animal studies (Mineur et al., 2023; Mineur and Picciotto, 2019; Pang et al., 2016; Jiang et al., 2016; Wilson and Fadel, 2017). The use of nicotine has been demonstrated to both increase and decrease anxiety, in various contexts (Kutlu and Gould, 2015; Mineur et al., 2023; Mineur et al., 2016; Picciotto, 2003). People report self-medicating with nicotine products to alleviate anxiety (Ferraz Lima et al., 2023), and there is an association between genes of the nicotinic receptor system and anxiety (Gillentine et al., 2018; Markett et al., 2011). LYNX2, a nicotinic modulatory protein, is encoded by the *Lynx2*/*Lypd1* gene, expressed in key regions of anxiety and fear circuitry, such as the amygdala and medial prefrontal cortex, in addition to other CNS sites (Dessaud et al., 2006; Tekinay et al., 2009; Sherafat et al., 2021), and sites outside the nervous system (Song et al., 2024; Omori et al., 2024). The LYNX2 protein binds to and reduces the activity of nAChRs (Tekinay et al., 2009; Wu et al., 2015) in response to its natural ligand, acetylcholine, as well as the drug of abuse, nicotine. The *Lynx2* null mutant mouse (*Lynx2*KO) displays heightened fear and anxiety-like behavior across several paradigms of basal anxiety, including light–dark box, elevated plus and social interaction tests (Sherafat et al., 2021; Tekinay et al., 2009), and changes in an acoustic startle response in male *Lynx2*KO mice (Sherafat et al., 2021). Removal of *Lynx2* leads to hyperactivity of nAChR responses, sensitizing nAChR to agonist and slowing receptor desensitization (Tekinay et al., 2009). We posited that an imbalance of nicotinic signaling is playing a role in the heightened basal anxiety-like behavior, which can influence situational or specific threats in the environment (O’Leary et al., 2013). The development of affective disorders often results from long-term adaptation to dynamic and complex conditions (such as basal anxiety and context). Therefore, we sought to probe how heighted nAChR activity in anxiety-related circuits (Calhoon and Tye, 2015; Anderson and Adolphs, 2014) might contribute to longer-term adaptive responses in Lynx2 KO mice, employing fear extinction and social defeat stress behavioral paradigms.

Neural mechanisms allow the encoding of salient events into memories by associative learning processes, and these help us respond later to cues that may predict a threat. This can be experimentally assessed by a fear conditioning paradigm that pairs an innocuous conditioned stimulus, such as a tone, with an unconditioned stimulus, such as a shock (Hegedüs et al., 2022). Since these processes employ coincidence detection to encode a suite of associated events into memories, however, not all encoded cues are predictive. New experiences which thwart conditioned expectations allow us to update learned associations that might be coincidental and not causative. Extinction of conditioned fear is an active learning process that produces a new association, integrating the conditioned association into a safety memory and is accompanied by a reduction in a conditioned fear response (Bouton et al., 2021; Lissek et al., 2004; Grillon, 2008; Craske et al., 2014; Furini et al., 2014). Cholinergic neurons respond to unexpected sensory cues that play a role in fear learning and extinction (Hegedüs et al., 2022; Herry et al., 2008; Crimmins et al., 2023). Elevations in nAChR signaling in the amygdala have been shown to inhibit fear extinction, whereas nAChR inhibition there promotes it (Jiang et al., 2016), underscoring the need of regulating nAChR activity. Fear extinction provides insight into the complex interactions that basal state of anxiety impacts on learning and responses to a specific threat.

Social stress is a pervasive form of stress experienced by most animals and is a known component of many human affective disorders such as depression and anxiety (Fiksdal et al., 2019; Krishnan et al., 2007). An etiologically relevant paradigm of conflict for the rodent species is the chronic social defeat stress test (CSDS), where test mice undergo daily defeat sessions characterized by both physical bouts and extended sensory contact with an aggressor mouse (Golden et al., 2011; Hultman et al., 2016; Bagot et al., 2016). CSDS is a highly validated assay that produces two divergent stress responses in social interaction tests, resilient, approaching the aggressor mouse, versus susceptible, avoiding it (Berton et al., 2006; Lagace et al., 2010; Golden et al., 2011; Hultman et al., 2018; Bagot et al., 2016; Hultman et al., 2016). The resilience response to CSDS has been shown to increase upon nicotine exposure via a mechanism involving BDNF, a trophic factor implicated in many neuroplasticity processes (Khalifeh et al., 2020; Mallei et al., 2019; Pagliusi et al., 2018). It has also been shown that CSDS elicits BDNF release in the ventral tegmental area (VTA) in susceptible wild-type mice (Koo Wook et al., 2016; Koo et al., 2019). While the VTA, a midbrain dopaminergic structure, is not considered a part of the primary anxiety-related circuit, it has an important role in many processes, including assessing the difference between expectation and outcome, and neuroplastic changes underling it (Anacker et al., 2016; Wang et al., 2023; Cao et al., 2010).

We probed the extent to which the dysregulation of nAChRs in anxiety-related circuits and subsequent heightened basal anxiety in *Lynx*2 KO mice would alter the complex behaviors of fear extinction and social defeat (Tekinay et al., 2009; Sherafat et al., 2021). Our prediction was that their heightened baseline anxiety-related phenotype would disproportionately affect their response to fear extinction and social defeat. To begin to address the mechanism, we studied the effect of *Lynx2* removal on nAChR signaling and any subsequent changes in BDNF.

## Materials and methods

### Animal model

All animal procedures were conducted in accordance within the guidelines of Lehigh University IACUC to ensure the humane and ethical treatment of the animals. Animals were kept on a 12/12-h light–dark cycle with ad libitum access to food and water. Animals were weaned at 21 days of age and separated by sex into group housing ranging from 2 to 5 animals per cage. A total of 3- to 8-month-old naïve, male and female, wild-type (WT) (C57BL/6 J), *Lynx2*KO, and double null mutant mice for the α7 nAChR and *Lynx2* (α7/*Lynx*2 double KO mice) were used. Breeding of the *Lynx2*KO mice included null mutant crosses to create knockout mice for experiments, backcrosses the C57BL/6J mice (C57) to maintain genetic diversity and avoid genetic drifts, and crosses of *Lynx2* heterozygous mice from the backcrosses to other *Lynx2* heterozygous mice from different pairs or knockout mice to refresh to the null mutants. The backcross was introduced every three generations. The α7/*Lynx2* double KO mice were created by crossing *Lynx2*KO null mutant mice with α7 null mutant mice. Litters from first generation double heterozygous (*Lynx2* +/− α7 +/−) mice were crossed together, and second-generation mice were genotyped to find either one allele as a null mutant (−/−) and the other allele as heterozygous (+/−) or for double mutants (D−/− or D+/−). Combinations of the possible genotypes [such as *Lynx2*KO (−/−), *α*7 heterozygous (+/−) and *Lynx2* heterozygous (+/−) and α7KO (−/−)] were crossed until several double mutants were verified. The double mutants were crossed to create mice for experiments. Backcross to C57BL/6 J mice were added every three generations and added into the breeders as described before to avoid genetic drift. All mice were genotyped using a polymerase chain reaction assay from DNA that was extracted from a tail biopsy. Each tissue sample was genotyped 2–3 times before the confirmed genotype was assigned. All mice were involved in one behavioral assay.

### Statistics and graphs

Power analysis was conducted before data collection. For all statistical analyses, a *α* = 0.05 was used to establish a statistically significant result.

For fear extinction experiments, data were collected as the number of seconds spent freezing during the behavior trial on day 1 and day 2 of extinction. This was divided by the total amount of time in the chamber and multiplied by 100% to obtain the % freezing on each day. These data are represented by line graphs. A two-tailed paired Student’s *t*-test was performed on the set of observations on day 1 of extinction and the set of observations on day 2 of extinction in order to determine if there was a significant reduction in freezing. As a measure of fear extinction, the percentage freezing on day 2 was subtracted from the percentage freezing on day 1. Thus, the reduction in % freezing could be compared between groups such as sex and genotype. This was done using the R anova_test() function to compute one- or more-way ANOVA, depending on the number of variables being evaluated for their effect. *Post-hoc* Student’s *t*-tests were conducted when a significant result was obtained. These datasets are represented by boxplots. For the 5-day extinction trials shown in 1b, these line graphs were simply extended to have 5 days on the x-axis. A repeated measures ANOVA was performed on the set of observations to determine whether there was a significant reduction in freezing across days.

For chronic social defeat stress experiments, data were collected as seconds spent in the interaction zone before and after the novel mouse is added. Time spent in the interaction zone with a C57 stranger mouse versus the CD1 aggressor is graphed on a boxplot with individual data points. SI ratios were calculated by dividing the time in the interaction zone with a stranger present by the time in the interaction zone with no stranger present. SI ratios are shown on a boxplot with a scatter plot overlaid. Paired two-tailed Student’s *t*-tests were performed on the SI ratios between the C57 baseline interaction and the CD1 aggressor interaction after CSDS. Unpaired two-tailed Student’s *t*-tests were performed to assess the effect of genotype on SI ratios at each time point. Mice were assigned as either resilient or susceptible based on the SI ratio of a certain trial. Fisher’s exact test was performed on the distribution among these groups.

Western blots were scanned using a ChemiDoc imaging system using the stain-free gel program and analyzed using ImageJ. Molecular weight of bands was determined by dividing the location of each band on the standard by the length of the gel. This was plotted on the y-axis against the logarithm of the known molecular weights of the standards. A line of best fit could be generated from this scatter plot. Then, the quotient associated with the distance traveled along the gel of the sample bands can be fit to this line, the x-value solved for, and the exponent of this value determined. The relative intensity of BDNF in samples as compared to actin can be determined by taking the pixel intensity of each band, as well as the space directly above and below it as a control. The intensity of the BDNF band was divided by the intensity of the actin band, and these values were plotted on a bar graph, with error bars representing the standard error among the digital measurements. No statistical analysis was performed on this gel since only one replicate of the gel was run.

All major behavioral assays were performed with a minimum of three replicates or independent cohorts to ensure a biological phenomenon as opposed to a one-time result. Replicates were temporally separated tests, and each included a different set of wild-type and knockout mice. All data are given as 95% confidence intervals (CIs) and are represented as AVG ± SEM. *p < 05, **p < 0.01, and ***p < 0.001.

### Fear conditioning

Fear conditioning and extinction is a 3-day experiment carried out in a conditioning hub (Coulbourn Instruments) where mice were placed inside an isolation cubicle (Coulbourn Instruments) to prevent ambient light and sound interference. The hub environment was lined with silver metallic walls, washed with isopropyl alcohol between trials, and had a mounted shock floor. On day 1, mice were placed in the cubicle and underwent a 2-min acclimation which consists of 2, 30 s sound (80 dB and 8 kHz) presentations, 2 min apart (20 WT mice, male; *n* = 10, female, *n* = 10; 19 *Lynx2* KO mice, male, *n* = 9; female, *n* = 10). To induce fear conditioning, during the last 2 s of the sound, the animal received a mild (0.5 mA) foot shock, with a 30-s rest period after the last foot shock, and the animals were returned to their home cage. For the weaker fear extinction paradigm, mice received only 1 tone-shock pairing at 0.25 mA shock. This was run with a different set of mice than the 2 tone-shock pairing. Fear conditioning is a day of learning for mice, and they learn to expect that the shock will follow when a tone is heard.

### Fear extinction

After the first day of fear conditioning, day 2 and 3 fear extinction took place, with multiple measures taken to control for context. Mice were placed in a different cubicle than the one used for day 1 of fear conditioning, the walls of the hub were switched to colorful patterns, a lavender scent was introduced, and between trials, the cubicle was washed with the novel cleaning agent, ethanol. This creates a different environment for the mouse and leaves the tone as the only constant. After 30 s of acclimation in the extinction chamber, the animal was presented with 3 sound presentations at the same frequency and intensity as on the training day, each 30 s long and 2 min apart, but with no foot shocks.

The behavior of the mice was recorded using an in-hub camera, and time spent freezing was analyzed using Coulbourn FreezeFrame software. The data are collected based on freezing, which was considered to be no bout of movement in a 2-s frame. A minimum cutoff value of 50% freezing on day 1 was used to eliminate mice that did not show freezing behavior. The criteria for an extinguished fear response were a statistically significant decrease in percent freezing to tone (CS) between day 2 (Ext 1) and day 3 (Ext 2).

To confirm data, we extended the test from 2 days to 5 days following the same procedures. Mice were placed in a different cubicle than the one used for day 1 of fear conditioning, the walls of the hub were switched to colorful patterns, a lavender scent was introduced, and between trials, the cubicle was washed with the novel cleaning agent, ethanol. This creates a different environment for the mouse and leaves the tone as the only constant. After 30 s of acclimation in the extinction chamber, the animal was presented with 3 sound presentations at the same frequency and intensity as on the training day, each 30 s long and 2 min apart, but with no foot shocks.

### Fear conditioning and extinction with pharmacological treatment

Fear conditioning was performed as previously described but with the addition of an IP injection given to *Lynx2*KO mice and WT mice 1 h prior to fear extinction on days 2 and 3. Mecamylamine (Mec) (a non-specific nAChR blocker), Methyllycaconitine (MLA) (an α7 antagonist), and Dihydro-β-erythroidine (DHβE, a β2* nAChR antagonist) can cross the blood–brain barrier at several documented doses, with enough potency to produce changes to nAChR function (Turek et al., 1995; Damaj et al., 1995). Doses were chosen based upon such studies. Mec (0.3–2.5 mg/kg, Abcam, Cambridge, MA), DHβE (2 mg/kg, Tocris Bioscience, Bristol, UK), and MLA (5 mg/kg, Sigma – Aldrich, St Louis, MA) were dissolved in 0.9% saline. The control animals were injected with 0.9% saline. Successful fear extinction was defined as a decrease in percent freezing from Ext 1 to Ext 2. Ext 2 is analyzed as the final extinction time point.

### Light–dark box

The light–dark box assay was conducted in TruScan motion tracking arena (Coulbourn Instruments). A dark walled box was placed inside the back half of the arena with the front half surrounded by clear walls. An arched opening was made between the two areas. *Lynx2*KO or WT mice were initially placed in the light side, and their location was monitored for 10 min by the TruScan software. Mice show an increase in anxiety-like behavior when spending an extended period in the dark causing an increased latency. For pharmacological studies, mice were given an injection, IP, 1 h prior to the start of testing.

### Chronic social defeat stress

CSDS followed a method adapted from the standardized protocol (Golden et al., 2011), with the CD1 mouse strain used as aggressors (Hsieh et al., 2017). First, CD1 mice were singly housed for 7 days followed by a screen for aggression over 3 days. During each day of screening, an 8 to 20-week-old WT male mouse was added to the home cage of the CD1, and the latency of the CD1 to attack is recorded. The WT mouse was removed upon attack or after 3 min. An attack was defined as a physical altercation that included, but was not limited to, biting, shoving, rushing, jumping onto tail rattling, and kicking. CD1 aggressors must show aggression in at least 2 subsequent sessions and have an attack latency of less than 60 s to be considered as an aggressor.

We set up two groups: non-defeated and defeated mice. Non-defeated mice (WT, *Lynx2*KO, α7/Lynx2double KO (α7/L2 double KO) were housed with littermates after weaning. They were placed in the same room as defeated mice and not disturbed until they were used for social interaction. For defeat, test mice (WT, *Lynx2*KO, α7/*Lynx2*double KO) were exposed to a CD1 aggressor mouse for a daily bout of 10 attacks. The attacks were limited to 10 to prevent excessive harm to the test mice that was observed with a defined time window. Following the bout, the intruder mouse was physically separated from the CD1 aggressor within the CD1 home cage but kept in sensory contact for 24 h. This was repeated each day for 10 days, after which, the test mice were singly housed for 24 h to await behavioral testing.

### Acute social defeat stress

We adapted the 10-day CSDS to test the robustness and sensitivity of the post-defeat phenotypes in this particular genetic model of anxiety-like behavior. We carried out the same procedure as described above in CSDS, but the defeat sessions occurred over 3 days. After the 3rd day of defeat, the test mice were singly housed. We found that the 3 days were sufficient to induce enough of a response in the test mice that conferred with the matching behavioral results of the 10-day protocol.

### Social interaction

The social interaction (SI) test occurred 24 h after the defeat session ended and mice were singly housed. SI ratios were assessed in Coulbourn TruScan motion tracking arenas. The arena was calibrated with an interaction zone against the back-most wall, and a wire containment cage was built to the specifications of the protocol (Golden et al., 2011).

For each phase of social interaction test, post-defeat mice or controls (naïve WT and *Lynx2*KO mice that did not undergo defeat and were kept in the standard housing of 2–5 littermates), called defeated or non-defeated test mice, respectively, were placed in the center arena and tracked for 150 s. The initial phase consisted of an empty interaction zone cage. Immediately following the completion of phase 1, the test mouse was removed from the arena and returned to its home cage. During the break, a novel CD1 stranger mouse was added to the wire containment cage within the interaction zone. The same test mouse was again placed in the center of the arena and tracked for 150 s. During this second phase, sensory information could be transmitted from the stranger to the test mouse, but there was no physical contact.

The location of the test mouse was tracked by the TruScan software. Social interaction ratios were determined based on time in the target zone (24 × 14 × 9 cm box surrounding the interaction zone) with and without the stranger mouse present. The social interaction (SI) ratio was used to assign the test mouse to a group: resilient or susceptible. The SI ratio is calculated by the following formula:


$$SI=\frac{\text{Time}\;\text{spent}\;\text{in}\;\text{interaction}\;\text{zone},\text{stranger}\;\text{present}}{\text{Time}\;\text{spent}\;\text{in}\;\text{interaction}\;\text{zone},\text{empty}\;\text{zone}}$$

For these studies, an SI ratio above 1.5 was considered “resilient” or “approach” and below 1.5 “susceptible” or “avoidant” rather than the standard 1 as the *Lynx2*KO mice were shifted toward higher SI ratios. We choose an SI ratio of 1.5 as our cutoff to increase the criteria needed to reach the resilient phenotype. Upon separating the groups, the data are presented as the percentage of time spent in the interaction zone with the stranger mouse present.

Test mice first underwent a CD1 social interaction test with a CD1 stranger mouse on day 11. The stranger mice used in the social interaction were either naïve to the test mouse, or in some cases for the CD1, the stranger was the most remote CD1 aggressor to that test mouse, from day 1 of CSDS. Only males were used in these studies due to the difficulty of initiating attack behavior directed toward female mice (Takahashi et al., 2017).

### Co-immunoprecipitation (co-IP)

WT, *Lynx2*KO, and α7KO mice were anesthetized with isoflurane and rapidly decapitated. The BLA was bilaterally dissected using visual landmarks and immediately homogenized in the bullet blender tissue homogenizer (NextAdvance, Averill Park, NY, USA), with Co-IP buffer (50 mM Tris, 150 mM NaCl, 0.75% Triton-X 100, Pierce protease inhibitor cocktail). Dynabeads A (Thermo Fisher Scientific, Waltham, MA, USA) were pre-incubated with 5 μg rabbit anti-lypd1 (LYNX2) primary antibodies (Thermo Fisher Scientific, Waltham, MA, USA) and thoroughly washed. An input sample of each homogenate was removed and kept at −80 degrees Celsius until Western blot analysis. Brain homogenates were incubated at a concentration of 13 mg/mL with the Dynabeads-antibody complex for 3 days nutating at RT. After washing, LYNX2 (LYPD1) protein complexes including interacting proteins were eluted and immediately prepared for Western blot analysis. Input (sample prior to the immunoprecipitation) and supernatant (after the immunoprecipitation) lane were to be compared to IP samples in the Western blot analysis. Co-immunoprecipitation experiments were performed in three replicates, each with one *Lynx2*KO mouse and two WT mice for a total of 18 technical replicates with 9 animals. The *Lynx2*KO mice were used as a negative control for the LYPD1 antibody. The α7KO mice were used as a negative control for the α7 antibody (data not shown).

### Western blot analyses

Samples were denatured in 1x sample buffer (Thermo Fisher Scientific, Waltham, MA, USA) at 95°C and run on a 4–20% gradient SDS-PAGE gel (Bio-Rad Laboratories, Hercules, CA, USA). Gels were transferred onto activated PVDF membrane via a semi-dry transfer. The membrane was blocked with 5% milk/0.05% Tween-PBS for 1 h at 4°C followed by an incubation overnight at 4°C in mouse monoclonal anti-nicotinic acetylcholine receptor α7 Subunit (1:1000, Sigma-Aldrich, St Louis, MO) or rabbit polyclonal anti-BDNF (5 μg/mL, Millipore, Burlington, MA, USA) for the BDNF study. After thorough washing, membrane was incubated with conjugated goat anti-mouse (Abcam, Cambridge, MA, USA) at 1:10,000 for 2 h at 4°C or 1:10,000 or conjugated goat anti-rabbit (Abcam, Cambridge, MA, USA) at 1:10,000 for 2 h at 4°C for BDNF study. Membranes were incubated in ECL (Thermo Fisher Scientific, Waltham, MA, USA) and exposed to film. Loading controls were run using mouse anti-actin primary antibodies (Abcam, Cambridge, MA, USA) at 1:1000 dilution and goat anti-mouse secondary antibodies (Life Technologies, Carlsbad, CA, USA) at 1:40,000 dilution.

## Results section

### The loss of *Lynx2*, in a *Lynx2*KO mouse, leads to a robust lack of fear extinction

**Figure 1:**
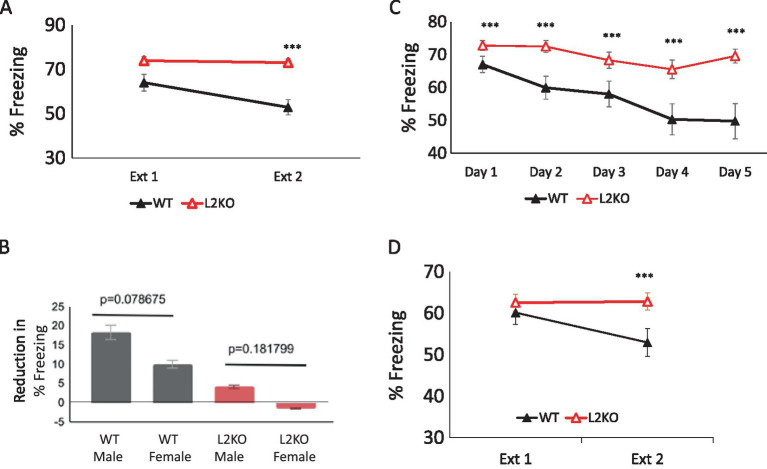
Marked lack of fear extinction in *Lynx2*KO mice. **(A)** Over the course of fear extinction, *Lynx2KO* mice (red line) do not undergo fear extinction as demonstrated by the maintenance of the degree of freezing between extinction day 1 and day 2. WT mice (black line) do undergo fear extinction, decreasing freezing over the course of 2 extinction days (*p* = 0.011, one-way repeated ANOVA). The *Lynx2*KO mice show higher amounts of freezing on both extinction days 1 (**p < 0.01) and 2 (***p < 0.001) as compared to WT mice. Y-axis is the % freezing to tone (CS) during the test period. **(B)** No sex differences were observed in fear extinction tests (*p* = 0.0787). The reduction in freezing behavior from day 1 to day 2 was 8.334 +/− 1.180% in WT males (*n* = 9) and 10.031 +/− 0.852% in females (black, *p* = 0.078675, *n* = 10). In L2KO males, the reduction in freezing was 4.149+/− 0.788% (*n* = 8), and in females, there was an increase in freezing of −1.467 +/− 0.910 (red, *p* = 0.182, *n* = 11). Y-axis is the reduction in percentage of time freezing during the test periods. **(C)** Extending extinction trials to 5 days did not ameliorate the extinction deficit in *Lynx2*KO mice. When extending the number of extinction days from 2 to 5, WT mice (black line) display a reduction in percent freezing to tone/CS while *Lynx2*KO mice (red line) have an overall maintenance of fear. *Lynx2*KO mice continue to display elevated freezing during extinction across all days (***p < 0.001). **(D)** With a weaker training protocol to mitigate any differences in fear learning between WT and L2KO mice, there were no significantly different levels of freezing (%) on test day 1 (Ext 1), (*p* = 0.542). By the second test day (Ext 2), the *Lynx2*KO mice (red line) still freeze more as compared to WT mice (black line), exhibiting a lack of fear extinction behavior (*p* < 0.001). WT *n* = 10, L2KO *n* = 10, Y-axis is percent freezing to tone (CS).

To discern how *Lynx2*KO mice responded in a paradigm that requires the updating of previously learned associations in response to new information, we tested the *Lynx2*KO in a fear extinction paradigm. Mice were trained by being subjected to two pairings of an innocuous tone and a foot shock and, 24 h later, were tested for freezing behavior in response to the tone alone (CS). Extinction trials were presented over 2 days, three CS trials per day. Over the course of fear extinction, *Lynx2*KO mice (red line) do not undergo fear extinction as demonstrated by the maintenance of freezing over the 2 extinction days. WT mice (black line), on the other hand, undergo fear extinction, measured as a reduction in freezing from extinction day 1 to day 2. A one-way repeated measures ANOVA was conducted, and there was a significant effect of genotype [*p* = 0.011, Wilks’ Lambda = 0.766, *F*_(1, 34)_ = 7.918]. Two paired *t*-tests were used for *post-hoc* comparisons. There was significantly greater freezing in *Lynx2*KO (73.93 +/− 1.84%) as compared to WT mice (68.75 +/− 2.81%) on day 1 of extinction (paired samples *t*-test, t (35) = 35.07, *p* < 0.001). On day 2 of extinction, the *Lynx2*KO mice (73.04 +/− 1.66%) also showed greater freezing than WT (58.88 +/−2.2822%), paired samples *t*-test, *t*(35) = 32.757, *p* < 0001. WT *n* = 20, L2KO *n* = 19, WT 95% CI [51.548, 64.224], L2KO 95% CI [69.536, 76.310], Cohen’s d = 1.535. Two-way ANOVA for sex*genotype *F* = 0.743, *p* = 0.395 (Figure 1A).

Comparison of sex differences demonstrated comparable amounts of extinction in the WT (*p* = 0.182) and *Lynx2*KO (*p* = 0.183) groups (Figure 1B), two-way ANOVA for gender genotype (*n* = 46, *p* = 0.511, F = 0.743, *p* = 0.395). These findings indicate that sex did not significantly influence the outcomes in this study, and the males and females were grouped. In the WT group, male extinguished fear 8.32 +/−0.18% (black, *p* = 0.078675, males *n* = 9), whereas WT females reduced freezing 10.03 +/− 0.85% (*n* = 10). *Lynx2*KO males extinguished 4.149 +/− 0.788%, and females increased their fear, extinguishing −1.4672 +/− 0.9105% (red, males *n* = 8, females *n* = 11).

We found that *Lynx2*KO mice displayed heightened fear responses and a lack of fear extinction, as demonstrated by a maintained level of freezing over the course of extinction trials. Fear extinction is an active process that results in the formation of a new memory in which the conditioned stimulus (CS) is no longer predictive of the unconditioned stimulus (US). We sought to distinguish between lack of ability to extinguish versus a delayed or reduced sensitivity, as it is possible the *Lynx2*KO mice need additional input to form a safety memory that can outcompete the original fear memory. To establish the strength and longevity of the abnormal response in the fear extinction paradigm of *Lynx2*KO mice, we extended the number of fear extinction sessions from 2 days to 5 days.

WT mice (black line) display a reduction in percent freezing to tone/CS over the 5 extinction days, while *Lynx2*KO mice (red line) demonstrate a maintenance of fear. Although *Lynx2*KO mice show a trend in inter-trial (within extinction day) extinction after several sound presentations, the reduction in freezing from day to day is not significant resulting in a lack of extinction behavior. A one-way repeated measures ANOVA was conducted to compare the effect of genotype on freezing during extinction day 1 through extinction day 5. There was a significant effect of genotype [*p* = 0.006, Wilks’ Lambda = 0.410, *F*_(4, 24)_ = 4.654]. Five paired *t*-tests were used for *post-hoc* comparisons, and in each day, the *Lynx2*KO group demonstrated significantly greater freezing than the WT one (day 1 *t*(28) = 50.919, *p* < 0.001, day 2, *t*(28) = 33.236, *p* < 0.001, day 3, *t*(28) = 28.178, *p* < 0.001, day 4, *t*(28) = 21.075, *p* < 0.001, day 5, *t*(28) = 20.199, *p* < 0.001 WT *n* = 12, L2KO *n* = 16. Day 5 95% CI: WT [53.230, 60.804], KO [67.828, 71.665], Cohen’s d = 1.089) (Figure 1C).

A possible confound in assessing extinction ability could be the heightened fear learning exhibited by *Lynx2*KO mice. To mitigate this, a milder training protocol used to establish the fear-based associative memory was implemented, using a single tone-shock pairing and reduced shock intensity. With this milder training protocol, there are no differences between groups in fear learning, as demonstrated by day 1 percent freezing to tone (CS). *Lynx2*KO mice (red line), however, continue to exhibit a lack of fear extinction behavior, whereas WT mice undergo fear extinction (black line). A one-way repeated measures ANOVA was conducted to compare the effect of genotype on freezing during extinction day 1 and extinction day 2. There was a significant effect of genotype [Wilks’ Lambda = 0.706, *F*_(1, 17)_ = 7.095, *p* = 0.016]. Two paired *t*-tests were used for *post-hoc* comparisons. A first paired samples *t*-test indicated no significant effect of genotype on day 1 freezing [*t*(18) = 31.983, *p* = 0.542]. A second paired samples *t*-test indicated a significant effect of genotype on day 2 freezing (*t*(18) = 24.055, *p* < 0.01. WT *n* = 10, L2KO *n* = 10, WT 95% CI [44.074, 61.840], L2KO 95% CI [57.740, 67.904], Cohen’s d = 0.996) (Figure 1D).

### Pharmacological blockade of α7 nAChRs restores fear extinction in *Lynx2*KO mice

**Figure 2:**
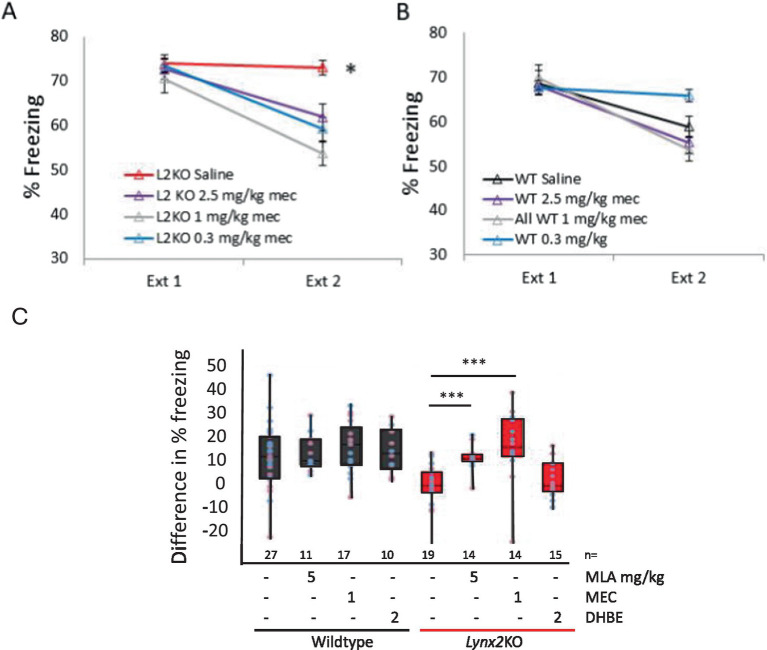
Cholinergic blockade implicates distinct nAChRs in *Lynx2*-mediated behaviors **(A)** Blocking nAChR channels with mecamylamine rescues the lack of fear extinction in Lynx2KO mice, compared to *Lynx2*KO mice without mecamylamine at all doses tested (red line, *p* = 0.001). Y-axis is percent of the time freezing to tone (CS). **(B)** Mecamylamine blockade of nAChRs does not have a significant effect in the WT mouse (*p* = 1.057). Y-axis is percent of the time freezing to tone. **(C)** Antagonism of α7, but not DHBE, nAChRs rescues the fear extinction phenotype in *Lynx2* KO mice (red, *p* = 0.0000186, two-way ANOVA). Blocking α7 nAChRs with MLA (1 mg/kg) allowed *Lynx2* KO mice (red) to extinguish, as seen as a reduction in freezing behavior (*p* = 0.000143). Each dot represents the difference in percent freezing (Ext 1 - Ext day 2) for a male (blue) or female (pink) mouse. A two-way ANOVA revealed that the difference in fear extinction among treated wild-type mice (black) was not statistically significant from naive wild-type mice. (*p* = 0.79) Y-axis is the reduction of freezing from day 1 to day 2.

We hypothesized that the *Lynx2*KO phenotype was due to nAChR hyperactivity from our current understanding of the mechanism of action of *Lynx2* and family members such as *Lynx1* (Tekinay et al., 2009; Ibañez-Tallon et al., 2002; Miwa et al., 2006; Sherafat et al., 2021; Morishita et al., 2010). We tested the ability of nAChR pharmacological treatment to restore normal extinction behavior in *Lynx2* KO mice (Figure 2A). Injection of mecamylamine, a general nAChR pore blocker, prior to extinction testing rescued fear extinction behavior in the *Lynx2*KO mice (*p* = 0.000413, unpaired Student’s *t*-test). These doses of mecamylamine, 0.3, 1.0, and 2.5 mg/kg, have no effect on the WT mice in this paradigm. A one-way repeated measures ANOVA was conducted to compare the effect of the drug in *Lynx2*KO mice on extinction, demonstrating a significant effect of the drug [Wilks’ Lambda = 0.750, *F*_(1, 56)_ = 6.236, *p* = 0.001]. Bonferroni *post-hoc* showed a significant effect at each mecamylamine dose; L2KO vs. L2KO 0.3 mg/kg (M = −7.154, SE = 2.568) *p* = 0.044, L2KO vs. L2KO 1 mg/kg (M = 11.337, SE = 2.66) *p* < 0.001, L2KO saline *n* = 20, L2KO 2.5 mg/kg *n* = 11, *p* < 0.001, L2KO 1 mg/kg *n* = 14, L2KO 0.3 mg/kg *n* = 10. 95% CI, L2KO [69.536, 76.310], L2KO 2.5 mg/kg [53.717, 69.934], L2KO 1 mg/kg [47.831, 59.615], L2KO 0.3 mg/kg [53.184, 65.183] (Figure 2B).

Utilizing more specific nAChR antagonists, we found that MLA, an α7 antagonist, rescued the *Lynx2*KO fear extinction phenotype and demonstrated levels of extinction comparable to WT mice (naive *n* = 19, treated *n* = 14, *p* = 0.000143, unpaired Student’s *t*-test) (Figure 2C). Dihydro-*β*-erythroidine (DhβE), a heteromeric nAChR antagonist as opposed to the homomeric α7 nAChR subtype, did not alter extinction in *Lynx2*KO mice (naive *n* = 19, treated *n* = 6, *p* = 0.4311) (Figure 2C), suggesting that the *Lynx2*-based extinction phenotype is mediated by α7 nAChRs [one-way ANOVA *F*_(7)_ = 10.629, *p* < 0.001] (Drasdo et al., 1992; Damaj et al., 1995). A two-way ANOVA was conducted on these data, assessing extinction across sexes and genetic lines. This revealed that the difference in fear extinction among treated wild-type (black) mice was not statistically significant as compared to naive WT mice (*p* = 0.79), whereas this effect was significant in *Lynx2*KO mice (red) (*p* = 0.0000186) (Figure 2C). In addition, a Cohen’s d-test was used to determine the effect size of these treatments on *Lynx2*KO mice. The effect size of mecamylamine was 1.15, the effect size of MLA was 1.22, and the effect size of DhβE was 0.277.

### Abnormal response to chronic social defeat in *Lynx2*KO—a nearly uniform resilience response

To investigate how an etiologically relevant stressor influences responses of mice predisposed to anxiety, we conducted chronic social defeat stress (CSDS) and assessed by a social interaction test. We first ran a control social interaction test, without a stress component, to confirm the abnormal sociability-like behavior of the *Lynx2*KO previously reported in (Tekinay et al. (2009) and determine whether it had been maintained over the course of generations. In social interaction tests, a test mouse was added to an arena with a restrained conspecific stranger mouse which allowed for the flow of sensory information of the stranger without physical contact. The evaluation of the phenotype was conducted by generating an SI ratio of time spent in the interaction zone with the stranger mouse vs. when there was no stranger mouse present, using a cutoff of 1.5 (see Methods, data not shown). The naive *Lynx2*KO mice avoided the C57Bl6 stranger mouse to a greater degree than WT mice did. These observations were consistent with previous reports (Tekinay et al., 2009), confirming the affective phenotype while allowing the CD1 mice to remain novel until the start of the CSDS sessions. (Tekinay et al., 2009).

**Figure 3:**
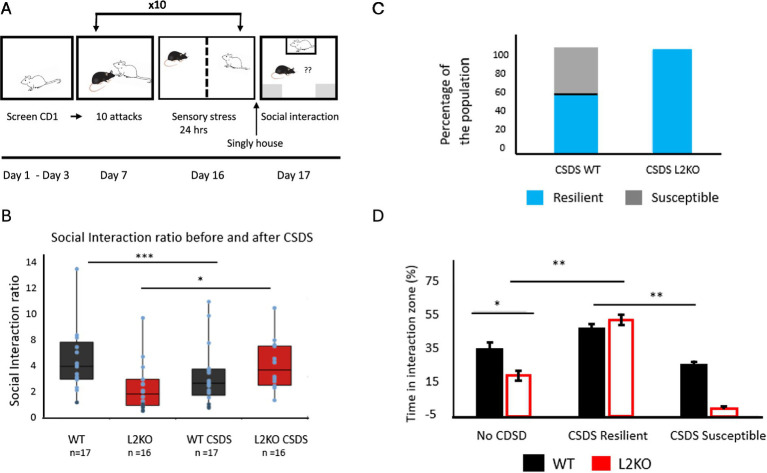
Shift in all social interaction behavior following CSDS in *Lynx2*KO mice. **(A)** Schematic of chronic social defeat stress timeline: Prior to CSDS, CD1 mice are screened for aggression. Mice undergo 10 days of CSDS (chronic social defeat stress), entailing daily bouts of aggressive interactions with extended sensory contact. Following the 10th day, mice are singly housed for 1 day before undergoing social interaction tests (SI). **(B)** After experiencing chronic social defeat stress, wild-type mice show a population split in social interaction phenotype between resilient (blue) and susceptible (gray) phenotypes in response to a CD1 mouse. All *Lynx2*KO mice, however, exhibit a population phenotype of resilient. **(C)** Prior to defeat, *Lynx2*KO mice display less time in the social interaction zone and exhibit social avoidance (No CSDS groups, *p* = 0.019). Based upon social interaction ratios post-defeat, there are WT mice that approach and spend a greater percentage of time in the interaction zone (CSDS Resilient) as well as WT mice that do not approach and spend less time in the interaction zone (CSDS Susceptible). There are no Lynx2KO mice that display a non-approach/susceptible phenotype after defeat. All *Lynx2*KO mice post-defeat spend a larger percentage of time in the interaction zone after defeat (*p* < 0.001). Y-axis is percentage of the cohort, resilient (blue) susceptible (gray). **(D)** Social interaction ratios (SI) change in WT (*p* < 0.001, *n* = 17) and KO mice post-defeat (*p* = 0.038, *n* = 17). Y-axis is SI ratio.

Having confirmed the social interaction phenotype was maintained in naive Lynx2KO mice, we conducted chronic social defeat stress (CSDS) on them. We performed 10 days of CSDS, entailing daily bouts of aggressive interactions with aggressive CD1 mice, with extended sensory contact between bouts. Following the 10th day, mice were singly housed for 1 day in a home cage before undergoing social interaction (SI) with a CD1 mouse as the stranger (Figure 3A). Measuring SI ratios following CSDS, the WT mice shift to lower values, whereas Lynx2KO mice demonstrate an increase in SI ratio, indicating an overall preference toward the stranger mouse (Figure 3B). Defeated WT test mice displayed the expected divergent stress responses of the population, with individuals displaying either the resilient/approach (blue) and susceptible/avoidant phenotypes (gray) (Figure 3C). On the other hand, all the post-defeat KO mice displayed the resilient/approach phenotype—a marked difference from pre-defeat scores, even with an SI ratio cutoff that increases the criteria for resiliency (Figure 3C). There was no significant difference in the time spent in the interaction zone by the resilient subset of WT mice and the Lynx2KO mice. There were significant differences in the time spent in the interaction zone between the resilient mice and the susceptible WT mice (Figure 3D). Based upon social interaction ratios post-defeat, there are WT mice that approach and spend a greater percentage of time in the interaction zone (CSDS Resilient) as compared to WT mice that do not approach and spend less time in the interaction zone (CSDS Susceptible). Interestingly, there were no Lynx2KO mice that display a non-approach/susceptible phenotype. All Lynx2KO mice post-defeat spent a larger percentage of time in the interaction zone. One-Way ANOVA F = 14.016, *p* < 0.001, Bonferroni post-hoc: WT vs. L2KO *p* = 0.019, WT vs. CSDS L2KO *p* = 0.005, L2KO vs. CSDS KO *p* < 0.001, L2KO vs. CSDS WT Resilient p < 0.001, CSDS WT Resilient vs. CSDS WT Susceptible p = 0.016, CSDS L2KO vs. CSDS WT Susceptible *p* < 0.001. WT *n* = 17, L2 KO *n* = 17, CSDS L2KO *n* = 17, CSDS WT Resilient *n* = 10, CSDS WT Susceptible *n* = 7. 95% CI: WT [27.383, 44.147], L2KO [12.504, 26.060], CSDS Resilient WT [24.782, 44.551], CSDS Susceptible WT [27.017, 43.841], CSDS Resilient L2KO [44.495, 61.701]. Cohen’s d WT No CSDS vs. KO NO CSDS = 1.118, No CSDS L2KO vs. CSDS Approach L2KO = 1.927 (Figure 3D).

**Figure 4:**
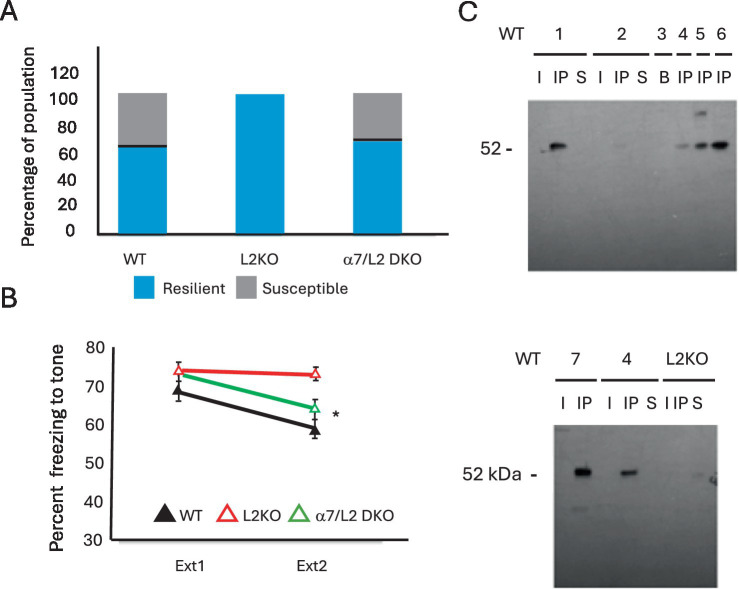
**(A)** In a 3-day acute social defeat stress paradigm, the WT and *Lynx2*KO mice continue to display the same trends of the 10-day CSDS paradigm: WT mice display a divergent response, while the *Lynx2*KO mice display one population response, resilience/approach. The double knockout restores the population response to that of WT levels with instances of both the susceptible and resilient phenotypes. **(B)** The α7/Lynx2 double knockout (green line) with a deletion of both the α7 nAChR and *Lynx2* genes restores fear extinction behavior to that of WT levels (black line) as compared to *Lynx2*KO mice (red line). A one-way repeated measures ANOVA was conducted to compare the effect of genotype on freezing during extinction day 1 and extinction day 2. There was a significant effect of the genotype on freezing; Wilks’ Lambda = 0.836 [*F*_(1, 49)_ = 4.793, *p* = 0.013]. Bonferroni *post-hoc*: WT vs. L2KO (M = 9.6752, SE = 3.095) *p* = 0.009, WT vs. α7L2 (M = 4.4857, SE = 3.22) *p* = 0.513, L2KO vs. α7L2KO (M = 8.3485, SE = 3.14) *p* = 0.032. WT *n* = 16, L2K *n* = 20, α7L2 *n* = 16. 95% CI: WT [51.548, 64.244], L2KO [69.536, 76.310], α7L2 [58.846, 69.045]. Cohen’s d L2KO vs. α7L2 = 1.050. **(C)** Western blot analyses showing the results of co-immunoprecipitation experiments. *Lynx2*KO was immunoprecipitated from homogenates of mouse amygdalae using an anti-*Lynx2*KO antibody. Then, a Western blot was run on these samples using anti-α7 nAChR antibodies (52 kd). These bands allow us to observe the stable complex of LYNX2 and the α7 nAChR subunit in the brain. WT animals are from different animals either of C57bl6 mice (WT 1-4) or WT littermates of the B2 nAChR-GFP mouse line (WT 5-7). WT 3 is from whole brain extract of a C57bl6 mouse, demonstrating enrichment of this complex in the amygdala. The amygdalae extracts of *Lynx2*KO mice were immunoprecipitated as controls (one shown here), Note that no bands are present in the *Lynx2*KO samples, indicating the specificity of the interaction.

Nicotine has been shown to augment resilience in response to CSDS (Khalifeh et al., 2020) and thus would be consistent with the nAChR hyperactivity that occurs due to *Lynx2* removal. The heightened basal anxiety in *Lynx2*KO mice could sensitize them to the defeat, in which case it might have been possible to detect both resilient and susceptible CSDS phenotypes in the KO cohort if given a reduced amount of defeat stress. Alternatively, the *Lynx2*KO mice could exhibit an exaggerated stress response that predisposes them to adopt a uniformly resilient phenotype independent of the amount of defeat stress. This may be analogous to the effects seen in fear extinction experiments in which the *Lynx2*KO mice failed to extinguish their fear, even with a lower training stimuli or an extended number of extinction days. To adapt to any potential hypersensitivity or responsiveness of *Lynx2*KO mice, we asked whether, by lowering the amount of stress given, some of the *Lynx2*KO mice would adopt a susceptible phenotype. We modified the CSDS paradigm by reducing the number of defeat days from 10 to 3, in an attempt to lower the degree of stress while retaining many of the critical features of the validated CSDS paradigm (Donahue et al., 2015; Pagliusi and Sartori, 2019; Qi et al., 2022). In this experiment, which we refer to here as acute defeat stress, mice were subjected to three trials per day on three consecutive days. In this 3-day modified CSDS paradigm, the *Lynx2*KO mice continued to display a resilient phenotype 100% of the time. WT mice display a divergent response including both resilience/approach and susceptible (Figure 4A).

### Genetic rescue of both fear extinction and social defeat stress in *Lynx2*KO mice via α7 nAChRs removal

α7 nAChRs have been shown to be one nAChR subtype targeted by the LYNX2 protein, although other nAChR subtypes have been shown to bind to LYNX2 (Pisapati et al., 2021; Wu et al., 2015; Tekinay et al., 2009). To confirm the pharmacological studies implicating α7 nAChRs in the fear extinction phenotype of *Lynx2*KO mice, we sought a genetic confirmation of α7 nAChRs involvement. The *Lynx2*KO mouse line was crossed to an α7 nAChR null mutant line (α7KO), to generate an independent *Lynx2*/α7 nAChR double KO line, and was tested using the fear extinction paradigm. The *Lynx2*/α7 nAChR double KO line was observed to extinguish to a similar degree as WT vs. *Lynx2*KO (*p* = 0.009), WT vs. *Lynx2*/α7 (*p* = 0.513), *Lynx2*KO vs. *Lynx2*/α7 (*p* = 0.032), rescuing the *Lynx2*KO phenotype, adding to the evidence that Lynx2-based extinction is α7-mediated. A one-way repeated measures ANOVA was conducted to compare the effect of genotype on freezing during extinction day 1 and extinction day 2. There was a significant effect of genotype on freezing; Wilks’ Lambda = 0.836 [*F*_(1, 49)_ = 4.793, *p* = 0.013]. Bonferroni *post-hoc*: WT vs. L2 KO (M = 9.6752, SE = 3.095) *p* = 0.009, WT vs. α7L2 (M = 4.4857, SE = 3.22) *p* = 0.513, L2KO vs. α7L2KO (M = 8.3485, SE = 3.14) *p* = 0.032*. WT *n* = 16, L2KO *n* = 20, α7L2 *n* = 16. 95% CI: WT [51.548, 64.244], L2KO [69.536, 76.310], α7L2 [58.846, 69.045]. Cohen’s d L2KO vs. α7L2 = 1.050 (Figure 4B). The effect size of this genetic modification was calculated as a reduction in percent freezing, in a Cohen’s d-test between the *Lynx2*KO and α7KO/*Lynx2* double KO lines. Within WT mice, we analyzed differences between males and females in line with reported observations for sex differences in fear extinction of WT mice (Clark et al., 2019). Within the entire dataset, we found a significant difference of genotype (ANOVA, *p* = 0.000799) and sex (*p* = 0.0248) but not in the sex × genotype interaction (*p* = 0.104). Sex differences were found in the WT group, but they were not found in the *Lynx2*KO or Lynx2/α7KO double KO groups. Male WT mice reduced freezing more than that of female WT mice (*p* = 0.01, male *n* = 10, female *n* = 13), contrasted with the *Lynx2* KO (*p* = 0.182) or *Lynx2*/α7 dKO groups (*p* = 0.267) (graph not shown).

We tested the Lynx2/α7KO double KO double knockout line in the 3-day acute defeat protocol. The α7/*Lynx2* double knockout mice displayed divergent stress responses of both susceptible and resilient phenotypes, to the same proportion as that of WT mice (Figure 4A), demonstrating that α7 nAChRs mediate these abnormal responses in *Lynx2*KO mice. These data support the involvement of *Lynx2* and α7 nAChRs in both fear extinction and acute social defeat paradigms.

### Physical interaction of LYNX2 and α7 naChRs in the amygdala

A LYNX2 protein-nAChR complex has never been detected *in vivo* but is a critical piece of information to interpret the functional relevance of the pharmacological and genetic results. To establish the physical interaction of LYNX2 and α7 nAChR proteins, co-immunoprecipitation experiments were performed to isolate LYNX2-nAChR stable complexes in the mouse brain. To detect specific components of the complex, we immunoprecipitated the LYNX2 protein from homogenates of the mouse amygdala using an anti-LYNX (LYPD1) antibody and performed Western blot analyses to detect the presence of α7 nAChRs using an anti-α7 nAChR antibody. Precipitated LYNX2-containing complexes from extracts of WT brain samples yielded a band corresponding to the α7 nAChRs subunit, providing evidence that LYNX2 forms a stable complex with α7 nAChRs in the mammalian brain (Figure 4C). No bands were seen in control samples prepared from *Lynx2*KO mice, indicating the specificity of the interaction. Taken together, our results indicate the role of *Lynx2* modulation of α7 nAChRs in the abnormal fear extinction and responses to social defeat stress.

### Defeat causes changes in *Lynx2*KO BDNF levels in the VTA

**Figure 5:**
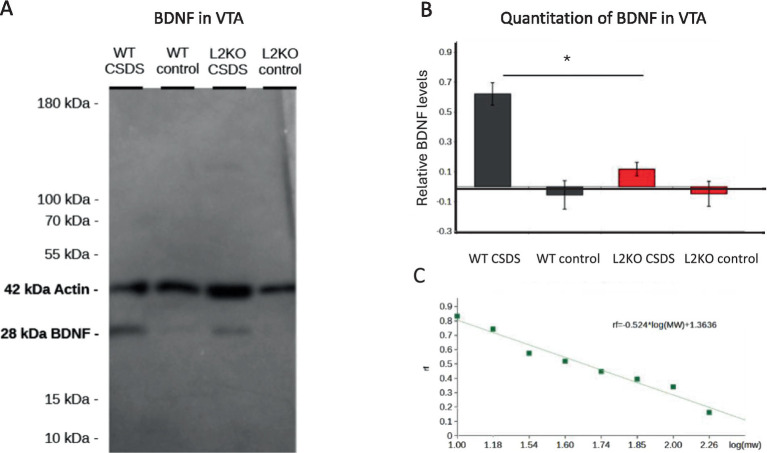
**(A)** BDNF levels in the VTA respond to the effects of CSDS: Western blot using anti-BDNF and actin antibodies showing the presence of BDNF in mouse VTA. **(B)** Quantitation of VTA BDNF levels: The relative intensities of the BDNF bands were normalized to actin, demonstrating a significant difference in BDNF protein between WT and *Lynx2*KO VTAs (*p* = 0.0149), measured using ImageJ software. The BDNF levels were significantly lowered after CSDS in WT. BDNF levels in the *Lynx2*KO VTAs did not change significantly. **(C)** The molecular weight of sample bands was determined by plotting the distance traveled by the standards against the log of their known molecular weight. A line of best fit was generated, and bands were fit to that line according to their rf. Bands were determined to have the molecular weight of approximately 41 and 28, corresponding to the expected values of actin and BDNF.

BDNF levels in the ventral tegmental area (VTA) have been shown to increase after CSDS in susceptible animals and to play a role in behavioral responses to CSDS (Koo Wook et al., 2016; Koo et al., 2019). We hypothesized that abnormalities in VTA BDNF levels might be a factor in the resilient phenotype seen in the *Lynx2*KO mice. We isolated the VTA from defeated mice and performed Western blots using an anti-BDNF antibody and quantitated the band intensity using Image J. There was a decreased BDNF level in *Lynx2*KO mice relative to WT controls, in both the naive and CDSD mice (Figure 5A). Compared to naive levels, there was an increase following defeat in both the groups but was more pronounced in the WT than in the *Lynx2*KO mice. Quantitation was obtained by measuring the intensity of BDNF normalized to actin of that lane, measured using ImageJ software (B2 CSDS M = 0.6213, SE = 0.0748; B2 control M = −0.0553, SE = 0.0949; *Lynx2* CSDS M = 0.1177, SE = 0.0461; *Lynx2* control M = −0.0477, SE = 0.0837. B2 CSDS vs. *Lynx2* CSDS *p* = 0.0149) (Figure 5B). The bands were found to have molecular weights of approximately 41 kDa and 28 kDa, corresponding to the expected values for actin and BDNF, respectively (Figure 5C). These data are consistent with the role of VTA BDNF in susceptibility.

## Discussion

*Lynx2* removal has been shown to lead to nAChR hyperactivity and heightened basal anxiety-like behavior (Tekinay et al., 2009). In this context, here we explored how augmented anxiety-like behavior may influence adaptive behaviors involving more specific threats. We found that *Lynx2*KO mice were unable to extinguish conditioned fear in extinction assays and showed a marked uniform resilience response in acute and chronic defeat stress. Fear extinction could be ameliorated through pharmacological blockade of α7 nAChRs, and in addition, fear extinction and acute social defeat could be restored through genetic perturbations of α7 nAChRs. Together with biochemical studies indicating a stable direct interaction of LYNX2 and α7 nAChRs in the amygdala, these data indicate that LYNX2 plays a regulatory role in α7 nAChR function for moderating fear extinction and responses to social defeat stress. Basal anxiety-like behavior in mice is thought to mirror trait anxiety in humans (O’Leary et al., 2013). There is a close relationship between trait anxiety, a person’s predisposition toward anxiety, and state anxiety, their reaction to specific cues or during specific events (Ree et al., 2008). Hence, it would be expected that a mouse model of elevated basal anxiety-like behavior may also have abnormalities in other fear and anxiety-like processes.

The cholinergic system is recognized as an important regulator of affective health (Mineur et al., 2016). There are optimal levels of ACh that can be beneficial, while excessive cholinergic signaling can be detrimental and lead to affective disorders. The cholinergic system is involved in both acquisition and extinction of fear memories (Kutlu and Gould, 2015), and acetylcholine is released to the auditory cortex in response to shock to promote fear learning (Krabbe et al., 2018). Extinction of learned fear involves plasticity mechanisms and the formation of new safety memories (Hascoët et al., 2001; Jiang et al., 2016; Kim et al., 2007; Dalton et al., 2012; Maren, 2015; Saha et al., 2017; Zhu et al., 2017) and includes, among other mechanisms, long-term depression of connections involved in the conditioned fear association, through activation of pathways coming from the medial prefrontal cortex to the amygdalar region (Cho et al., 2013; Gilman et al., 2018). mEPSCs frequency has been shown to be elevated in the prefrontal cortex of *Lynx2*KO mice in response to nicotine (Tekinay et al., 2009), and it would be interesting to test whether abnormalities in long-term depression are operating in *Lynx2*KO mice. Alterations in calcium levels by LYNX2 have been reported *in vitro* (Wu et al., 2015). Nicotinic receptor activation has been associated with lack of fear extinction (Kutlu and Gould, 2014), and optogenetic activation of cholinergic afferents from the medial prefrontal cortex into the amygdala can retard fear extinction, while optogenetic inhibition accelerates it (Jiang et al., 2016). The final output of fear comes from the central amygdala which integrates multiple inputs such as the prefrontal cortex. As such, increases in amygdala cholinergic tone may serve to weigh the balance in favor of maintenance of fear (Jiang et al., 2016). *Lynx2* is expressed in the prefrontal cortex and amygdala, among many other brain regions, so it is not possible to know to what degree each might contribute to the extinction phenotype (Tekinay et al., 2009; Sherafat et al., 2021). A regulatory mechanism such as LYNX2 that can dampen nAChR responsiveness may be one such control mechanism that allows the individual to appropriately update conditioned fears.

Our data point to a regulatory role of α7-containing nAChRs within key circuits underlying social defeat as well, in line with reports tying the nicotinic receptor system to modulation of social defeat (Ortiz et al., 2022; Khalifeh et al., 2020). A relationship between responses to social defeat stress and fear extinction has been demonstrated previously, when the WT subgroup that displayed the resilient/approach response to acute social defeat also displayed a lack of fear extinction (Meduri et al., 2013). In line with this profile, *Lynx2*KO mice exhibit an extreme form of this relationship, a marked lack of fear extinction and a uniform resiliency/approach following CSDS (Jasnow et al., 2004; Jasnow et al., 2005), likely a consequence of unbalanced nicotinic receptor activity.

A resilient social defeat stress response has been demonstrated in nicotine exposed animals (Khalifeh et al., 2020), inducing brain-derived neurotrophic factor (BDNF), a trophic protein implicated in many neuroplasticity processes (Mallei et al., 2019; Khalifeh et al., 2020; Chou et al., 2014; Monkol et al., 2016). Other work has linked increased BDNF in the VTA to susceptible defeat outcomes (Koo Wook et al., 2016). Social defeat stress has been shown to be altered by dopaminergic activity in susceptible mice (Zhao et al., 2021; Numa et al., 2019; Krishnan et al., 2007) and to mediate mPFC activity radiating into the amygdala (Numa et al., 2019). It is unknown if increases in excitability that induce BDNF are altered in these or other brain regions of the *Lynx2*KO mouse due to activity-dependent acetylcholine release (Acquas et al., 1996; Letzkus et al., 2011). In our studies, α7 nAChRs have been implicated in the tests performed here, but it should be noted that the α7/*Lynx2* DKO was tested with the 3-day acute defeat stress paradigm and was not tested in the standard 10-day CSDS one, so the direct test of α7 subtypes in CSDS is still an open question. Lower baseline levels of VTA BDNF or reduced BDNF induction in response to social defeat stress may explain, in part, the resilient phenotype of *Lynx2*KO mice. The reduction in BDNF levels post-defeat was more substantial in WT mice, but given the low starting levels of BDNF in naive mice, the modest reduction of BDNF in the *Lynx2*KO VTAs might be due to a floor effect. These data are consistent with the reported role of VTA BDNF in susceptibility (Letzkus et al., 2011), although more work on this line needs to be done to clarify the relationship further.

We have focused this study on the relationship of basal anxiety to other anxiety-mediated behaviors. Because of expression of *Lynx2* in multiple brain regions, however, the phenotype of *Lynx2*KO mice is not confined to anxiety-like behavior. Interestingly, male *Lynx2*KO mice have been shown to have reduced acoustic startle response (Sherafat et al., 2021). Elevations in the startle response have been correlated with higher anxiety (Pantoni et al., 2020), so the reductions in acoustic startle in male *Lynx2*KO mice are not likely due to their increased basal anxiety-like behavior. The neural circuitry for acoustic startle response only partially overlaps that of basal anxiety, illustrating effects of *Lynx2* outside of the regions we have explored here.

In humans, individuals vary in their responses to social stress and later vulnerability to psychiatric disorders (Charney and Manji, 2004; Krishnan et al., 2007). Defining the biological factors that underlie predispositions toward anxiety and how environmental stressors act to modify these biological factors can aid in the development of effective therapies. Our results are not inconsistent with studies linking variation in the human LYNX2 gene with heightened levels of anxiety in humans (Anderson et al., 2024). The *Lynx2*KO mouse model adds to the framework that nicotinic receptors may be an important factor in understanding affective disorders. Genetic factors which predispose an individual to heightened basal anxiety-like behavior could impair appropriate responses to more specific stressors. These results have implications for the treatment of inextinguishable anxiety and fear, such as that in generalized anxiety disorders, post-traumatic stress disorder, phobias, and other severe disorders.

## Data Availability

Data available on request to authors.
